# Cooperation therapy between anti-growth by photodynamic-AIEgens and anti-metastasis by small molecule inhibitors in ovarian cancer

**DOI:** 10.7150/thno.41708

**Published:** 2020-01-16

**Authors:** Jun Dai, Min Xu, Quan Wang, Juliang Yang, Jinjin Zhang, Pengfei Cui, Wenwen Wang, Xiaoding Lou, Fan Xia, Shixuan Wang

**Affiliations:** 1 Department of Obstetrics and Gynecology, Tongji Hospital, Tongji Medical College, Huazhong University of Science and Technology, Wuhan 430030, China.; 2 Engineering Research Center of Nano-Geomaterials of the Ministry of Education, Faculty of Materials Science and Chemistry, China University of Geosciences, Wuhan 430074, China.

**Keywords:** anti-metastasis, anti-growth, aggregation-induced emission, small molecule inhibitors, photodynamic therapy

## Abstract

Metastasis is one of the main causes of death and treatment failure in ovarian cancer. Some small molecule inhibitors can effectively inhibit the metastasis of primary tumors. However, they do not kill the primary tumor cells, which may lead to continuous proliferation. Herein, we have prepared a multifunctional nanoparticles named TPD@TB/KBU2046, which consisted of three functional moieties: (1) KBU2046 (small molecule inhibitor) that can inhibit the metastasis of the primary tumors, (2) TB (photodynamic-AIEgens) that may suppress the growth of the primary tumors, and (3) TPD, which contains TMTP1 (a targeting peptide, which specifically binds to highly metastatic tumor cells) that can enhance the TB/KBU2046 dosage in the tumor site.

**Methods:** The TPD@TB/KBU2046 was prepared by nano-precipitation method. We linked the targeting peptide (TMTP1) to the nanoparticles *via* amidation reaction. TPD@TB/KBU2046 nanoparticles were characterized for encapsulation efficiency, particle size, absorption spectra, emission spectra and ROS production. The combinational efficacy in image-guided anti-metastasis and photodynamic therapy of TPD@TB/KBU2046 was explored both* in vitro* and* in vivo*.

**Results:** The TPD@TB/KBU2046 showed an average hydrodynamic size of approximately 50 nm with good stability. *In vitro*, TPD@TB/KBU2046 not only inhibited the metastasis of the tumors, but also suppressed the growth of the tumors under AIEgens-mediated photodynamic therapy. *In vivo*, we confirmed that TPD@TB/KBU2046 has the therapeutic effects of anti-tumor growth and anti-metastasis through subcutaneous and orthotopic ovarian tumor models.

**Conclusion:** Our findings provided an effective strategy to compensate for the congenital defects of some small molecule inhibitors and thus enhanced the therapeutic efficacy of ovarian cancer.

## Introduction

The ovarian cancer is a common gynecological malignant tumor with the highest mortality rate 1-2. As the early symptoms are not obvious and the diagnosis rate is low, most patients are clinically diagnosed as advanced stage 3-4. At present, clinical treatment of ovarian cancer is still surgical resection combined with chemotherapy, but the 5-year survival rate is less than 40% 5. Metastasis of ovarian cancer can occur *via* the transcoelomic, haematogeneous, or lymphatic route, and is responsible for the major obstacle to the success of treatment 6-7. Thus, inhibition of tumor metastasis is extremely important for ovarian cancer treatment.

Recently, many small molecule inhibitors, such as tea polyphenols, curcumin, KBU2046, BENC-511, ZD6474, *et al*, have been reported for inhibiting tumor metastasis 8-12. Among them, KBU2046 is an innovative small molecule inhibitor, which has significant anti-metastasis effect and opens up a novel strategy for cancer treatment. Xu et al. demonstrated that KBU2046 can be inserted very precisely into the fissures of heat shock protein 90 and cell division cycle 37 heterocomplex, thereby affect the binding of the client kinase protein RAF1 to the heterocomplex, resulting in limited cell motility. Although KBU2046 can effectively inhibit the metastasis of tumor cells, it has no cytotoxic effect. Similarly, some small molecule inhibitors such as MPT0G211, DSHN, BDP5290 and BMS-777607 *etc*. also have good effect in inhibiting metastasis of tumors, but no effect on cell viability 13-16. That is to say, even using these small molecule inhibitors, tumor cells can still proliferate *in situ*. The inherent properties of these small molecule inhibitors, including lack of cytotoxicity, make their biocompatibility excellent, but this is a defect in the treatment of tumors. Therefore, based on the defect of these small molecule inhibitors, it is necessary to introduce a therapeutic element to suppress the growth of the tumors.

Photodynamic therapy (PDT) is a burgeoning, noninvasive and safe therapeutic assay that utilized a photosensitizer, light of particular wavelength and oxygen to achieve its therapeutic effect 17-20. Photosensitizer is able to generate reactive oxygen species (ROS) upon light irradiation which can induce tumor cell death 21-24. As photosensitizer plays a critical role in undertaking the efficacy of PDT, the design and function of photosensitizer has been unremittingly improved over the decades 25-27. Traditional photosensitizer easily aggregates in aqueous phase due to the π-π stacking and rigid planar structures, for instance, porphyrin and its derivatives. Due to the aggregation-caused quenching effect, fluorescence quenching and ROS production efficiency reduction, the application of traditional photosensitizers has been greatly affected 28-30. Fortunately, Tang's group discovered the aggregation-induced emission luminogens (AIEgens), which provided a possibility to solve the issue of aggregation-caused quenching (ACQ) 31-34. The AIEgens have been developed as specific nanomedicine for cancer theranostics, such as image-guided tumor resection, cell tracking, monitoring of drug delivery processes and detection of biological processes 35-39. Importantly, some AIEgens with special structures can also be used as photosensitizers which possess high ROS production efficiency and strong fluorescence emission in aggregation state 40-43. The integration of brightness and photosensitization within a single AIEgen exhibits great convenience for therapeutic strategy with simplicity and reproducibility.

**Scheme 1:**
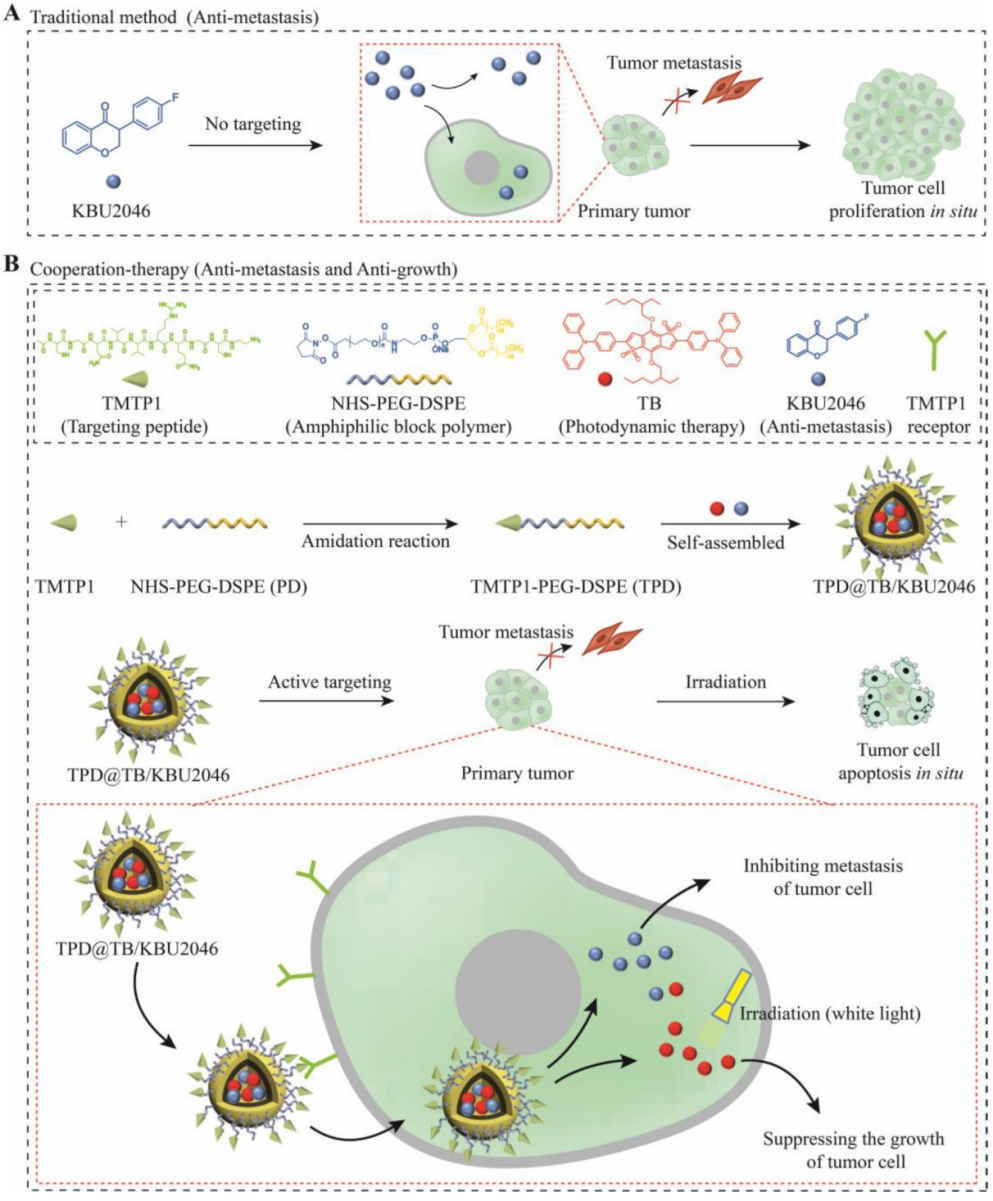
(**A**) Some small molecule inhibitors can effectively inhibit the metastasis of primary tumor; however, they were incapable of preventing tumor cells from continuously proliferating in situ. (**B**) Cooperative therapy had both anti-metastasis and anti-growth effect on tumor. Firstly, NHS-PEG-DSPE was conjugated with TMTP1 by amidation reaction to yield the TMTP1-PEG-DSPE. Then, the amphiphilic block polymer TMTP1-PEG-DSPE encapsulates KBU2046 and TB into the core of nanoparticles through hydrophobic self-assembly. The TPD@TB/KBU2046 nanoparticles were targeted and enriched in primary tumor by TMTP1 peptide. The KBU2046 in nanoparticles inhibited the metastasis of primary tumors. Moreover, TB entering tumor cells suppressed the growth of the primary tumor under white light irradiation.

Herein, we have successfully prepared a self-assembled nanoparticle (TPD@TB/KBU2046) containing small molecule inhibitor (KBU2046) and photodynamic-AIEgen (TB) (**Scheme 1**). In addition, TPD@TB/KBU2046 nanoparticles contain tumor targeting peptide (TMTP1), which specifically binds to highly metastatic tumor cells 44-47, thereby enhancing the accumulation of KBU2046 and TB in tumors. *In vivo*, our results showed that TPD@TB/ KBU2046 nanoparticles not only possess excellent tumor targeting, but also had cooperative effects of anti-metastasis and anti-growth, which provided a promising strategy for the treatment of ovarian cancer.

## Materials and Methods

### Materials

Dimethylsulfoxide (DMSO), tetrahydrofuran (THF), dimethyl formamide (DMF) was purchased from China National Medicines Co. Ltd (Shanghai, China). 1,2-Distearoyl-sn-glycero-3-phosphoethanolamine-N-[succinimidyl(polyethylene-glycol)-2000] (NHS-PEG-DSPE), 1,2-distearoyl-sn-glycero-3-phosphoethanolamine-N [methoxy(polyethylene glycol)-2000] (PEG-DSPE) were purchased from Xi'an Ruixi Biological Technology Co. Ltd (Xi'an, China). The peptide TMTP1 was purchased from GL Biochem Ltd (Shanghai, China). PVDF membrane was purchased from Millipore Corporation (Bedford, MA, USA). Fetal bovine serum was purchased from Gibco Company (Grand Island, NY, USA). 2′,7′-dichlorofluorescin diacetate (DCFH-DA) and Rose Bengal (RB) were provided by Yeasen Co. Ltd. (Shanghai, China). 9,10-Anthracenediyl-bis(methylene)dim alonic acid (ABDA) and 5-chloromethylfluorescein diacetate (CMFDA) were provided by Sigma-Aldrich (St Louis, MO, USA). Cell Counting Kit-8 (CCK8) was purchased from Beyotime Biotechnology (Shanghai, China).

### Synthesis of TMTP1-PEG-DSPE

1,2-Distearoyl-sn-glycero-3-phosphoethanolamine-N-[succinimidyl(polyethylene glycol)-2000] (NHS-PEG-DSPE, PD) was linked with TMTP1 (T) by amidation reaction to yield the TMTP1-PEG-DSPE (TPD). In brief, NHS-PEG-DSPE (10.00 mg, 3.4 μmol) was added to a DMF solution (1 ml) of TMTP1 (17.57 mg, 0.0170 mmol). The above solution was poured into ultrapure water and stirred for 24 h. Then, the reaction product was added to water solution and purified by dialysis (MWCO 3500) in flowing ultra-pure water for 48 h. After lyophilization, the product TPD was obtained with a yield of 73.4%.

### Preparation of TPD@TB/KBU2046 nanoparticles

NHS-PEG-DSPE (PD, 1.36 mg), TMTP1-PEG-DSPE (TPD, 0.64 mg), 2,6-Bis(4-(diphenylamino)phenyl)-4,8-bis((2-ethylhexyl)oxy)benzo[1,2-b:4,5-b']dithiophene 1,1,5,5-tetraoxide (TB, 0.8 mg), and 4'-fluoroisoflavanone (KBU2046, 0.8 mg) were simultaneously dissolved in tetrahydrofuran (THF) solution (1 mL). Sonicator with a microtip probe was used to stir the aforesaid solution for 5 min at 10 W. The mixed solution was added into the water, whereafter, the organic solvent THF in the mixed solution was blown dry with nitrogen. The solution was then filtered *via* a 0.2 μm filter membrane to get targeted nanoparticles. Nano-ZS ZEN3690 (Malvern Instruments) was used to detect the particle size at 25 °C. Particle size was measured in PBS buffer.

### Animal model

The experimental animal scheme strictly complied with the requirements of the experimental animal ethics committee of Tongji Medical College and conformed to the principles of animal protection, animal welfare, and ethics. BALB/c nude mice were purchased from HFK Bioscience Co. (Beijing, China). They were fed normally and provided unlimited access to water. Briefly, 1 ×10^6^ SKOV-3 or EGFP- SKOV-3 cells were subcutaneously inoculated into the right anterior side of female BALB/c nude mice. Tumor growth was measured using a vernier caliper. The tumor volume was calculated as: volume = 0.5× ((tumor length) × (tumor width) × (tumor width)).

### Anti-tumor effects of nanoparticles were studied using subcutaneous tumor model

When the volume of SKOV-3 tumor reached around 20 mm^3^, SKOV-3 tumor-bearing mice were randomly split into five groups. Mice were injected with TPD@TB/KBU2046 (2.0 mg/ml, 200 μL)), PD@TB/KBU2046 (2.0 mg/ml, 200 μL), TPD@TB (2.0 mg/ml, 200 μL), TPD@KBU2046 (2.0 mg/ml, 200 μL) and TPD (2.0 mg/ml, 200 μL) *via* tail vein, respectively. After 12 h, the nanoparticles were distributed into the tumor and then the tumor area was exposed to white light (200 mW cm^-2^) for 20 min. The weight and tumor volume of the mice were daily recorded. The tumors and major organs were collected for histological observation by standard hematoxylin & eosin and immunohistochemical staining.

### Anti-tumor effects of nanoparticles were studied using orthotopic ovarian cancer model

15 female BALB/C nude mice (5-week-old) were operated under anesthesia to expose the abdominal cavity, and the EGFP-SKOV-3 cells were transplanted into right ovarian capsule. TPD@TB/KBU2046 (2.0 mg/ml, 200μL), PD@TB/KBU2046 (2.0mg/ml, 200 μL), TPD@TB (2.0 mg/ml, 200 μL), TPD@KBU 2046 (2.0 mg/ml, 200 μL) and TPD (2.0 mg/ml, 200 μL) were injected into the tail vein when the primary tumor was about 200 mm^3^. Surgical treatment was performed after four consecutive injections of nanoparticles. After resection of the tumors, the abdominal cavity (including the surgical area) of mice were treated with white light irradiation (Intensity: 200 mW cm^-2^) for 20 min. After treatment, the recurrence and metastasis of tumors in mice were observed by IVIS.

## Results and Discussion

### Fabrication and characterization of TPD@TB/KBU2046

**Figure 1:**
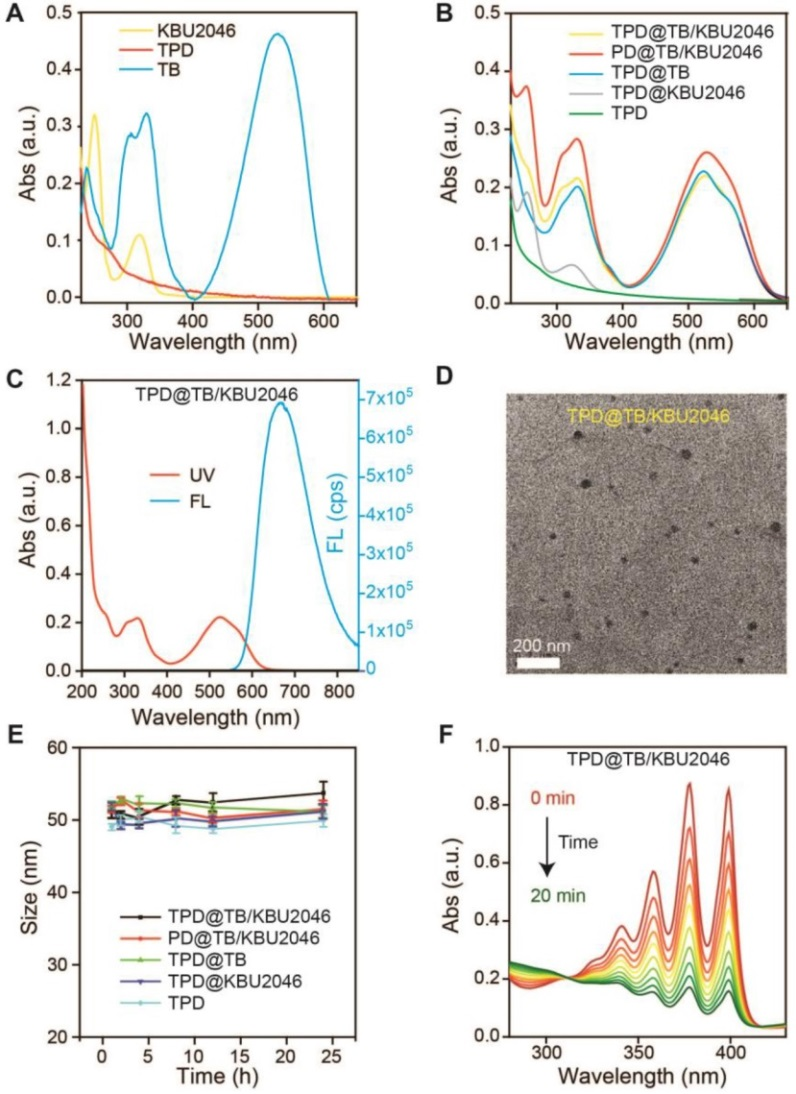
(**A**) UV-vis absorption spectra of TPD, TB and KBU2046. (**B**) UV-vis absorption spectra of TPD@TB/KBU2046, PD@TB/KBU2046, TPD@TB, TPD@KBU2046 and TPD. (**C**) UV-vis absorption and fluorescence spectrum of TPD@TB/KBU2046 (E_x_ =530 nm). (**D**) TEM image of TPD@TB/KBU2046. Scale bar: 200 nm. (**E**) Stability assay of TPD@TB/KBU2046 PD@TB/KBU2046, TPD@TB, TPD@KBU2046 and TPD in PBS for 24h at room temperature. (**F**) UV-vis absorption spectra of the miscible solution of ABDA and TPD@TB/KBU2046 upon light irradiation (white light, 100 mW cm^-2^).

According to our previous report, we synthesized the AIE-photosensitizer TPA-BDTO (TB) (Figure S1) 48. The fluorescence of TB increased with the increase of concentration (Figure S2A). At the same time, with the decrease of the THF/water ratio, TB gradually aggregated and its fluorescence increased (Figure S2B). These results indicated that TB was an AIEgen. The small molecule inhibitor KBU2046 was synthesized according to the literature (Figure S3) 12. PEG-DSPE (PD) was linked with TMTP1 by amidation reaction to yield the TMTP1-PEG-DSPE (TPD) (Figure S4/5) 49-50. UV-vis characteristic absorption spectra of TPD, TB and KBU2046 were shown in Figure 1A. KBU2046 and TB had two characteristic absorption peaks at 267 nm and 530 nm, respectively. The TPD@TB/KBU2046 was obtained *via* a modified nano-precipitation approach using TPD to form nanoparticles 51. The loading contents of TB and KBU2046 in TPD@TB/KBU2046 determined by UV-vis spectrophotometer were 24.20% and 21.94%, respectively. The loading contents of TB and KBU2046 in PD@TB/ KBU2046 were 26.21% and 24.47%, respectively. The loading content of TB in TPD@TB was 30.40% and the loading content of KBU2046 in TPD@KBU2046 was 22.06%. UV-vis absorption spectra of TPD@TB/ KBU2046, PD@TB/KBU2046, TPD@TB, TPD@KBU2046 and TPD nanoparticles were exhibited in Figure 1B. The TPD@TB/KBU2046 showed the main absorption peaks at 267 and 530 nm, which was in connection with KBU2046 and TB. The TPD@TB/KBU2046, PD@TB/KBU2046 and TPD@TB possessed a strong emission at 530 nm, while TPD@KBU2046 and TPD almost displayed no fluorescence (Figure 1C, Figure S7). Transmission electron microscopy (TEM) image confirmed the morphology and particle size of the TPD@TB/KBU2046 (Figure 1D). Importantly, the TPD@TB/KBU2046 in PBS solution displayed great stability within 24 h at room temperature (Figure 1E). The ROS indicator ABDA was used to confirm the ROS generation capability of TPD@TB/KBU2046, whose absorption decreased at 378 nm when ROS generated upon white light irradiation (Figure 1F). The intersystem crossing process between the lowest singlet excited state (S1) and triplet excited state (T1) of TB induced the ROS production. Comparing with the commercial photosensitizer (Rose Bengal), TPD@TB/KBU2046 possessed almost as good photosensitivity as it (Figure S8A/B). These results indicated that we have successfully prepared the TPD@TB/KB2046 nanoparticles, which had great stability and photosensitivity.

### Nanoparticles-mediated PDT inhibited the proliferation of SKOV-3 cells

**Figure 2:**
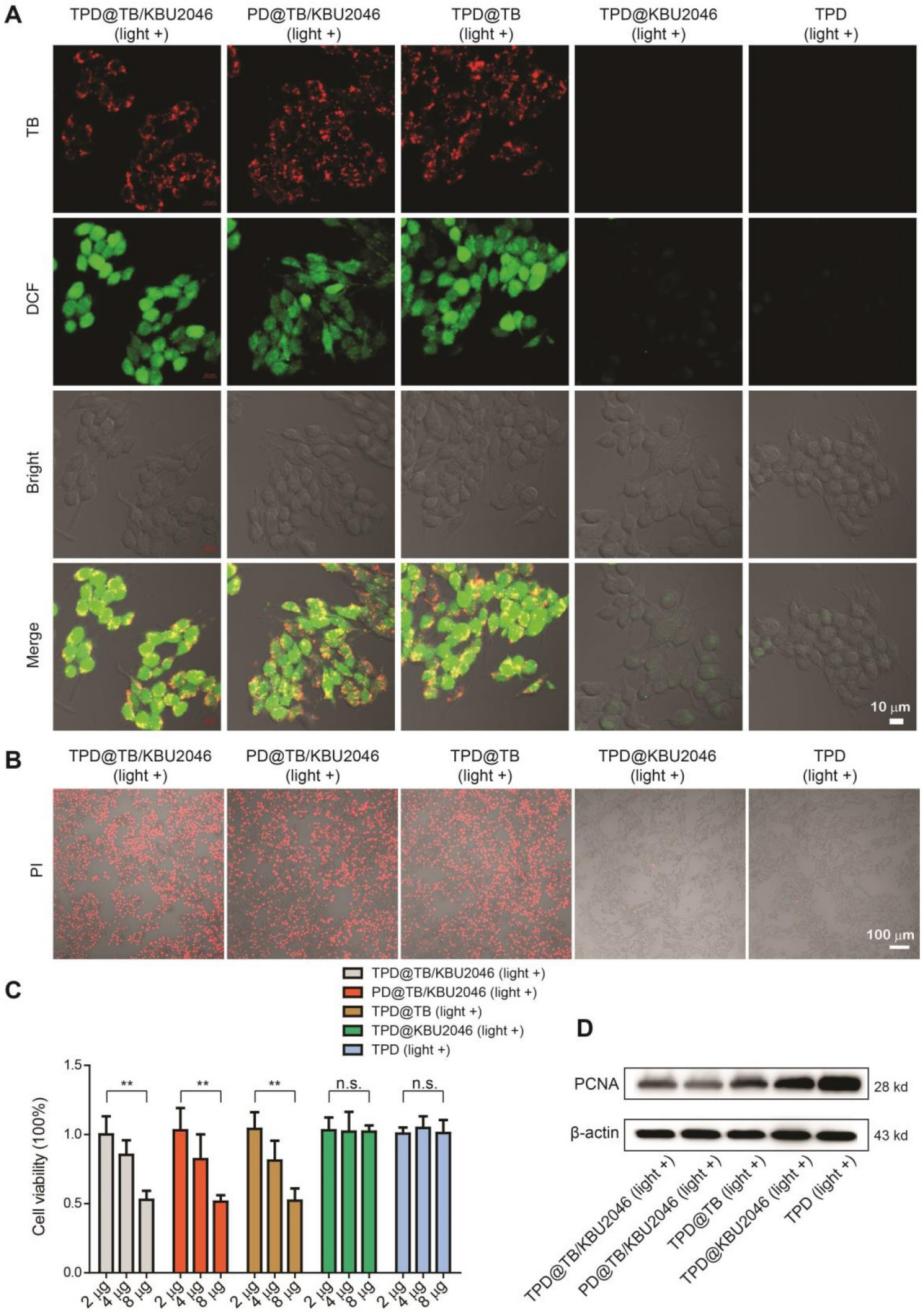
(**A**) SKOV-3 cells were treated with TPD@TB/KBU2046 (10 μg/ml), PD@TB/KBU2046 (10 μg/ml), TPD@TB (10 μg/ml), TPD@KBU2046 (10 μg/ml) and TPD (10 μg/ml), respectively, and then irradiated with white light (100 mW cm^-2^, 3 min). The ROS production in SKOV-3 cells was detected by DCFH-DA assay. Green fluorescence (DCF, E_x_ =488 nm, E_m_=505-540 nm); Red fluorescence (TPD@TB/KBU2046, PD@TB/KBU2046, TPD@TB, Ex: 488 nm, E_m_: 620-720 nm). Scale bar: 10 μm. (**B**) SKOV-3 cells were cultured with TPD@TB/KBU2046 (10 μg/ml), PD@TB/KBU2046 (10 μg/ml), TPD@TB (10 μg/ml), TPD@KBU2046 (10 μg/ml) and TPD (10 μg/ml), respectively, and then irradiated with white light (100 mW cm^-2^, 3 min), respectively. The dead SKOV-3 cells were detected by PI staining. Red fluorescence (PI, E_x_: 543 nm, E_m_: 620-680 nm). Scale bar: 100 μm. (**C**) SKOV-3 cells were incubated with different concentrations of TPD@TB/KBU2046, PD@TB/KBU2046, TPD@TB, TPD@KBU2046 and TPD nanoparticles and irradiated with white light (100 mW cm^-2^, 3 min), respectively. Then the viability of the SKOV-3 cells was evaluated by CCK-8 assay. Data were represented as mean ± SD (n =3). The results were analyzed *via* two-sided Student's t-test. ** P <0.01, n.s., not significant. (**D**) Expression of PCNA in SKOV-3 cells treated with TPD@TB/KBU2046, PD@TB/KBU2046, TPD@TB, TPD@KBU2046 and TPD upon light irradiation (white light, 100 mW cm^-2^, 3 min).

Five nanoparticles (TPD@TB/KBU2046, PD@TB/KBU2046, TPD@TB, TPD@KBU2046 and TPD) were respectively incubated with SKOV-3 cells for 4 h. Strong red fluorescence (TB) can be observed in the SKOV-3 cells by CLSM after cultured with TPD@TB/KBU2046, PD@TB/KBU2046 and TPD@TB (Figure 2A). After incubation with DCFH-DA and irradiation with white light, green fluorescence (DCF) was observed in the SKOV-3 cells in TPD@TB/KBU2046, PD@TB/KBU2046 and TPD@TB groups, which indicated that when TB was loaded into nanoparticles, it could enter SKOV-3 cells and produce excessive ROS under white light irradiation (Figure 2A). Furthermore, the viability of the SKOV-3 cells was evaluated using propidium iodide (PI) after cultured with TPD@TB/KBU2046, PD@TB/KBU2046, TPD@TB, TPD@KBU2046 and TPD upon light irradiation (Figure 2B). The numbers of the death cells in TPD@TB/KBU2046, PD@TB/KBU2046 and TPD@TB groups were more than that in TPD@KBU2046 and TPD groups, indicating that PDT effects were produced in TPD@TB/KBU2046, PD@TB/KBU2046 and TPD@TB groups. Meanwhile, the SKOV-3 cells viability was also evaluated by CCK-8 assay (Figure 2C). With the increase of the nanoparticle concentration in culture medium, the cell viability in TPD@TB/KBU2046, PD@TB/KBU2046 and TPD@TB groups decreased, which was in contrast with that in TPD@KBU2046 and TPD groups. The above results demonstrated that the TB loaded into nanoparticles can be used for image-guided PDT. To investigate the cytotoxic mechanism, we carried out western blotting to reveal the expression of proliferation factor proliferating cell nuclear antigen (PCNA) during the PDT process 52, 53. As shown in Figure 2D, the expression of PCNA in SKOV-3 cells decreased in TPD@TB/KBU2046, PD@TB/KBU2046 and TPD@TB groups, while it was strong in TPD@KBU2046 and TPD groups, which indicated that treating SKOV-3 cells with TB-loading nanoparticles could effectively reduce the expression of PCNA and inhibit cell proliferation under white light irradiation.

### Anti-metastasis efficiency of the nanoparticles *in vitro*

**Figure 3:**
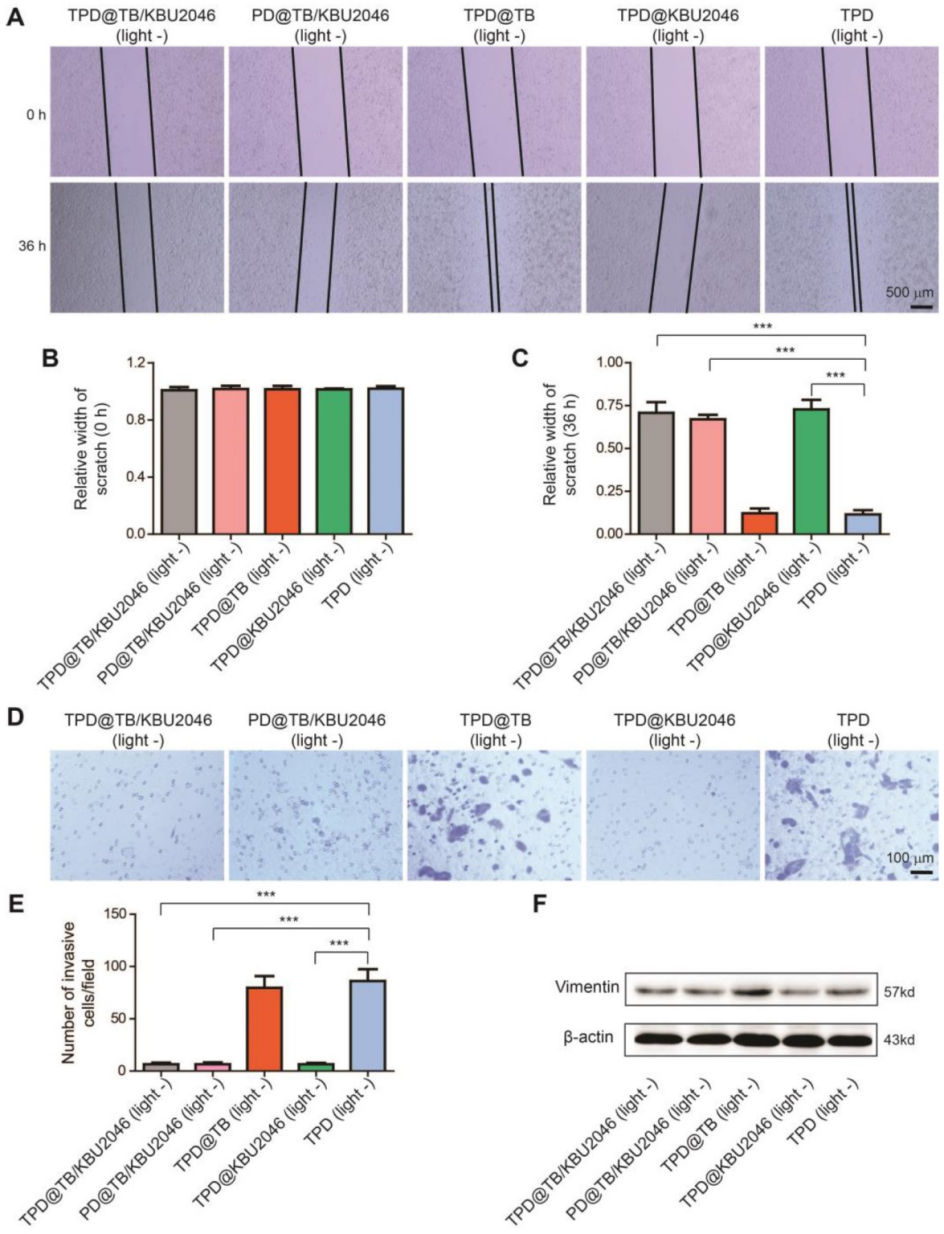
(**A**) The scratch width was measured and photographed at the beginning of the scratch test and 36 h later. Relative width of scratch was detected (**B**) at the beginning of the scratch test and (**C**) 36 h later. (**D/E**) Transwell assay was carried out to evaluate the metastatic potential of SKOV-3 cells after treated with TPD@TB/KBU2046 (10 μg/ml), PD@TB/KBU2046 (10 μg/ml), TPD@TB (10 μg/ml), TPD@KBU2046 (10 μg/ml) and TPD (10 μg/ml), respectively. The results were represented as mean ± SD (n =3). The data were analyzed *via* two-sided Student's t-test. *** *P* <0.001. (**F**) After 36 treated with TPD@TB/KBU2046 (10 μg/ml), PD@TB/KBU2046 (10 μg/ml), TPD@TB (10 μg/ml), TPD@KBU2046 (10 μg/ml) and TPD (10 μg/ml) for 36 h, the expression level of Vimentin in SKOV-3 cells was determined.

The effects of different nanoparticles on the migration capability of SKOV-3 cells were examined by cell scratch test. As pointed in Figure 3A/B, the width of the scratches in these five groups was basically the same at the beginning of the scratch test. However, 36 h after incubation, the scratch width of TPD@TB/KBU2046, PD@TB/KBU2046 and TPD@KBU2046 groups was significantly wider than that of the other two groups (Figure 3A/C). Moreover, the metastatic potential of the SKOV-3 cells was assessed by transwell assay after incubated with different nanoparticles, which represented that the numbers of the metastatic cells in TPD@TB/KBU2046, PD@TB/KBU2046 and TPD@KBU2046 were significantly less than that in the other two groups (Figure 3D/E). The scratch test and transwell assay confirmed that SKOV-3 cells treated with nanoparticles containing small molecule inhibitor KBU2046 had lower migration and metastasis capability. Vimentin was related to the metastasis of the tumor cells, and the higher its expression, the stronger its metastasis capability 54. Expression of Vimentin in SKOV-3 cells treated with TPD@TB/KBU2046, PD@TB/KBU2046 and TPD@KBU2046 nanoparticles was lower than that in other groups (Figure 3F). This result revealed that the small molecule inhibitor KBU2046-entrapped nanoparticles can inhibit tumor cell invasion and metastasis *via* Vimentin pathway.

### Biodistribution and tumor targeting of nanoparticles *in vivo*

**Figure 4:**
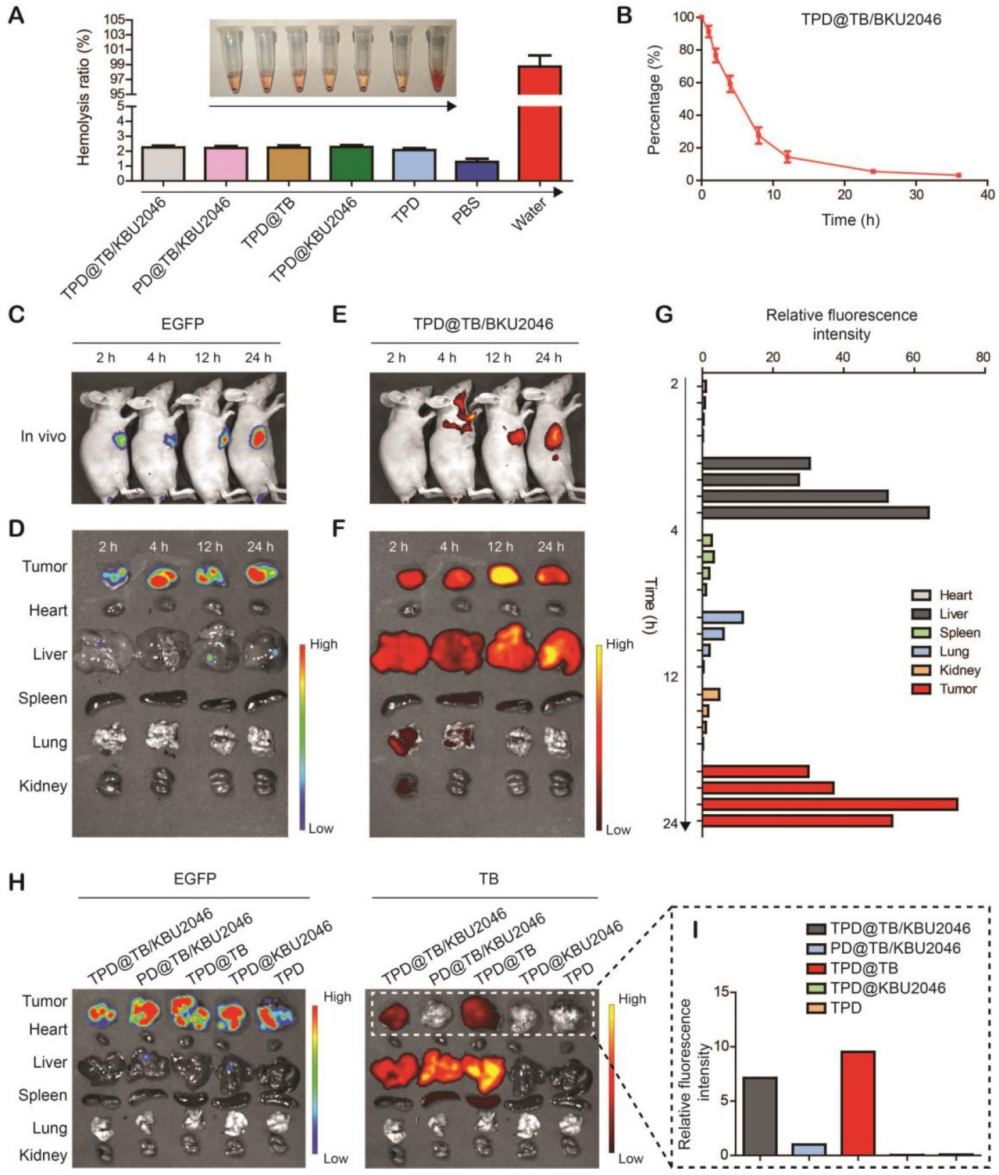
(**A**) Hemolysis assay after incubated with TPD@TB/KBU2046 PD@TB/KBU2046, TPD@TB, TPD@KBU2046, TPD, PBS (negative control), and ultrapure water (positive control) for 3 h in dark. The results were presented as the mean ±SD (n =3). (**B**) Circulation profile of TPD@TB/KBU2046 (2.0 mg/ml, 200 μl) in normal BALB/c female mice after injection *via* the tail vein. The results were presented as the mean ±SD (n =3). (**C/D**) EGFP imaging (E_x_=500 nm, E_m_=540 nm) of EGFP-SKOV-3 tumor-bearing mice after injection of TPD@TB/KBU2046 (2.0 mg/ml, 200 μl) for 2 h, 4 h, 12 h and 24 h *via* the tail vein. (**E/F**) Biodistribution of TPD@TB/KBU2046 (Ex =540 nm, Em =680 nm) in different organs and tumors in EGFP-SKOV-3 tumor-bearing mice after injected with TPD@TB/KBU2046 for 2h, 4h, 12h and 24 h *via* the tail vein. (**G**) The relative fluorescence intensity of each harvested tumors and organs were measured with a semi-quantitative biodistribution analysis. (**H**) Fluorescence images of EGFP and TB in different organs and tumors 48 h after the tail vein injection of TPD@TB/KBU2046, PD@TB/KBU2046, TPD@TB, TPD@KBU2046 and TPD, respectively. The coloured spectrum gradient bar indicated the fluorescence intensity. (**I**) Relative fluorescence intensity of each harvested tumors was measured with a semi-quantitative biodistribution analysis.

It was known that only the materials with hemolysis rate less than 5% can be applied to intravenous injection 55. Hemolysis test was used to assess the hemocompatibility of nanoparticles (Figure 4A). Negligible hemolysis happened in the five experimental groups as well as the phosphate buffered saline (negative control), however, lots of hemoglobin was observed in the ultrapure water (positive control). The hemolysis ratio of the five experimental groups was lower than 5%, which indicated that the nanoparticles were appropriate for intravenous injection. *In vivo*, the pharmacokinetics showed that TPD@TB/KBU2046 remained in the circulation for a relatively long time without non-specific scavenging, which was conducive to the enrichment of nanoparticles in tumors (Figure 4B). In order to confirm the targeting effect of nanoparticles *in vivo*, the SKOV-3 cell line expressing green fluorescent protein (EGFP-SKOV-3) was used to establish a tumor-bearing mice model, and then the tumors were localized according to the spontaneous fluorescence (Figure 4C/D). TPD@TB/KBU2046 gradually accumulated in tumors, especially at 12 and 24 h after injection, the fluorescence coincidence of EGFP and TB indicated that TPD@TB/KBU2046 had a good tumor targeting effect (Figure 4C/E). *Ex vivo*, fluorescence imaging showed that TPD@TB/KBU2046 was mainly concentrated in liver and tumor, and the fluorescence intensity of tumor reached the maximum at 12 h post-injection, which indicated PDT was appropriate at 12 h post-injection (Figure 4F/G). To further demonstrate the tumor targeting of nanoparticles, EGFP-SKOV-3 tumor-bearing mice were treated with TPD@TB/KBU2046, PD@TB/KBU2046, TPD@TB, TPD@KBU2046 and TPD, respectively. The biodistribution of EGFP and TB in the tumors and organs were detected by IVIS and was shown in Figure 4H/I. Due to the presence of TB in the nanoparticles, fluorescence was observed in the tumors and organs in TPD@TB/KBU2046, PD@TB/KBU2046 and TPD@TB groups, but not in TPD@KBU2046 and TPD groups. Further analysis showed that strong fluorescence signals were observed in tumors of TPD@TB/KBU2046 and TPD@TB groups, but weakness in the PD@TB/KBU2046 group (without TMTP1 peptide). These results suggested that nanoparticles with TMTP1 peptide exhibited excellent targeting efficacy in cancer therapy.

### Cooperation therapy between anti-growth and anti-metastasis to inhibit tumor growth and metastasis in subcutaneous tumor model

**Figure 5:**
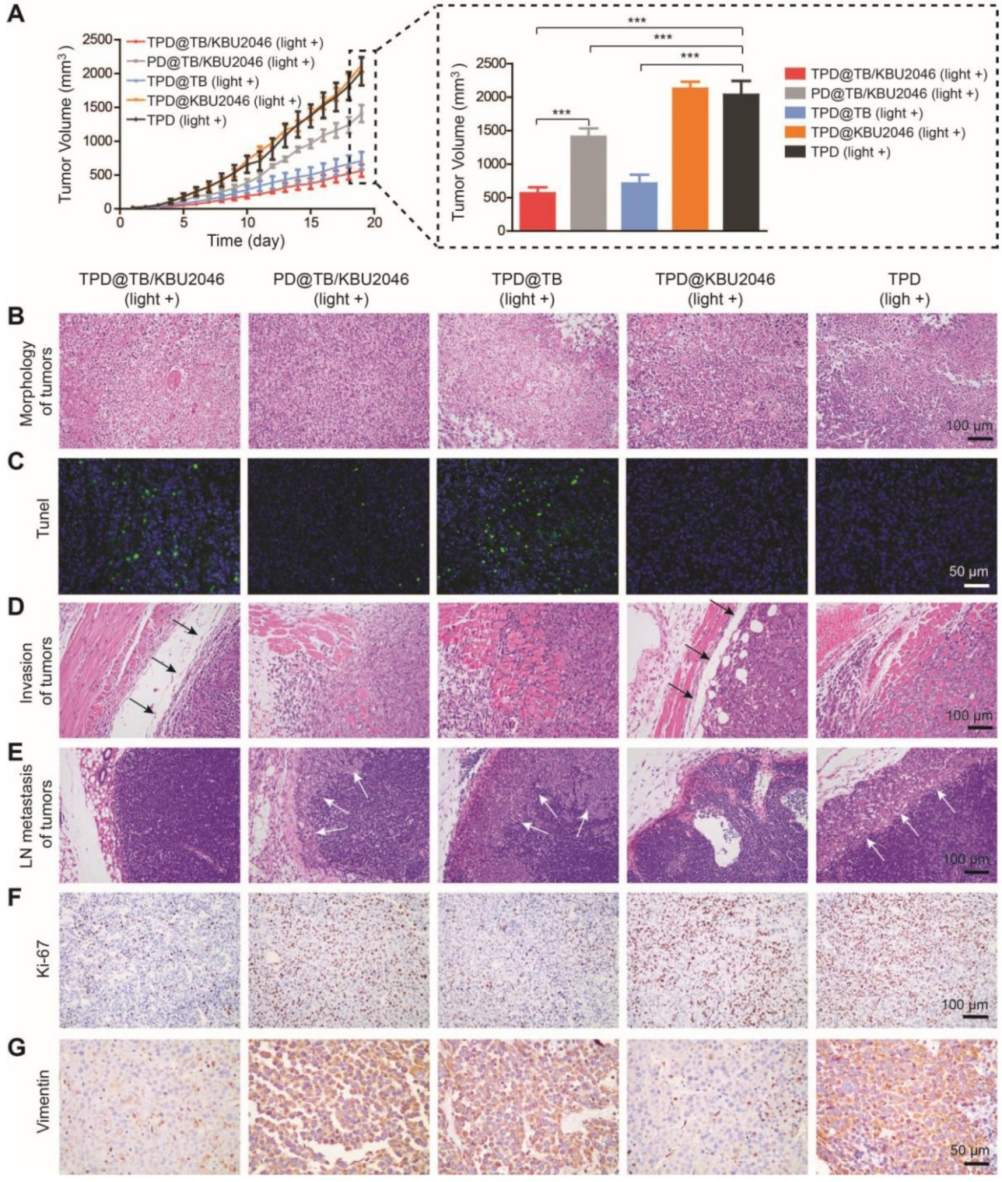
(**A**) The tumor growth curves of SKOV-3 tumor-bearing mice treated with TPD@TB/KBU2046 (light +), PD@TB/KBU2046 (light +), TPD@TB (light +), TPD@KBU2046 (light +) and TPD (light +), respectively. Irradiation intensity: 200 mW cm^-2^. Irradiation time: 20 min. Data were presented as the mean ± SD (n =5) and analyzed by two-sided Student's t-test. *** P <0.001. (**B**) H&E staining, (**C**) Tunel staining, (**D**) boundary (the black arrow showed a clear boundary of the tumor) and (**E**) lymph node metastasis (the white arrow showed metastatic tumors of lymph nodes) of the tumor in SKOV-3 tumor-bearing mice treated with the five different nanoparticles. IHC staining of (**F**) Ki-67 and (**G**) Vimentin in SKOV-3 tumor after treated with the five different nanoparticles.

In order to investigate the anti-tumor efficacy of the nanoparticles *in vivo*, we divided SKOV-3 tumor-bearing mice with tumor volume of approximately 20 mm^3^ into five groups: (1) TPD@TB/KBU2046; (2) PD@TB/KBU2046; (3) TPD@TB; (4) TPD@KBU2046; and (5) TPD. The mice in each group were exposed to white light. As shown in Figure 5A, the growth of the tumors in TPD@TB/KBU2046, PD@TB/KBU2046 and TPD@TB groups was both suppressed compared with TPD group. The inhibition rate of TPD@TB/KBU2046 group was 72.3%, that of TPD@TB group was 65.09%, and that of PD@TB/KBU2046 group was 30.81%. These results suggested that tumor growth can be significantly inhibited when TB and TMTP1 were simultaneously contained in nanoparticles. H&E staining revealed that tumor tissues in TPD, PD@TB/KBU2046 and TPD@KBU2046 groups were compact, whereas tumors in TPD@TB/KBU2046 and TPD@TB groups were sparse, and a large number of necrotic tumor cells could be found (Figure 5B). TUNEL staining exhibited abundant apoptotic tumor cells in TPD@TB/KBU2046 and TPD@TB groups, few apoptotic cells in PD@TB/KB2046 group, and almost no apoptosis in TPD and TPD@KBU2046 groups (Figure 5C). These results indicated that nanoparticles containing TMTP1 and TB developed superb PDT efficacy, thereby causing increased apoptosis/necrosis of tumor cells.

The anti-metastasis effect of nanoparticles was evaluated according to the tumor boundary and lymph node metastasis. The black arrow in Figure 5D showed a clear tumor boundary, suggesting that tumor cell in TPD@TB/KBU2046 and TPD@KBU2046 groups had a weaker invasive capability. Meanwhile, metastatic tumors (white arrow) appeared in the lymph nodes in the PD@TB/KBU2046, TPD@TB, and TPD groups, but not in the TPD@TB/KBU2046 and TPD@KBU2046 groups (Figure 5E). These results consistently implied that the nanoparticles contained both TMTP1 and KBU2046 inhibited tumor invasion and metastasis.

Ki-67 was a marker that reflected the proliferative capability of tumor cells 56. The numbers of Ki-67 positive cells in TPD@TB/KBU2046 and TPD@TB groups were significantly less than the other groups, indicating that targeted PDT significantly suppressed tumor cell proliferation (Figure 5F). Vimentin expression in tumors was significantly correlated with invasion and metastases. Vimentin was highly expressed in tumor in PD@TB/KBU2046, TPD@TB, and TPD groups, but lowly expressed in TPD@TB/KBU2046 and TPD@KBU2046 groups (Figure 5G). In conclusion, our results suggested that when the nanoparticles contained TMTP1, TB and KBU2046, can they achieve both anti-metastasis and anti-growth effects* in vivo*.

**Figure 6:**
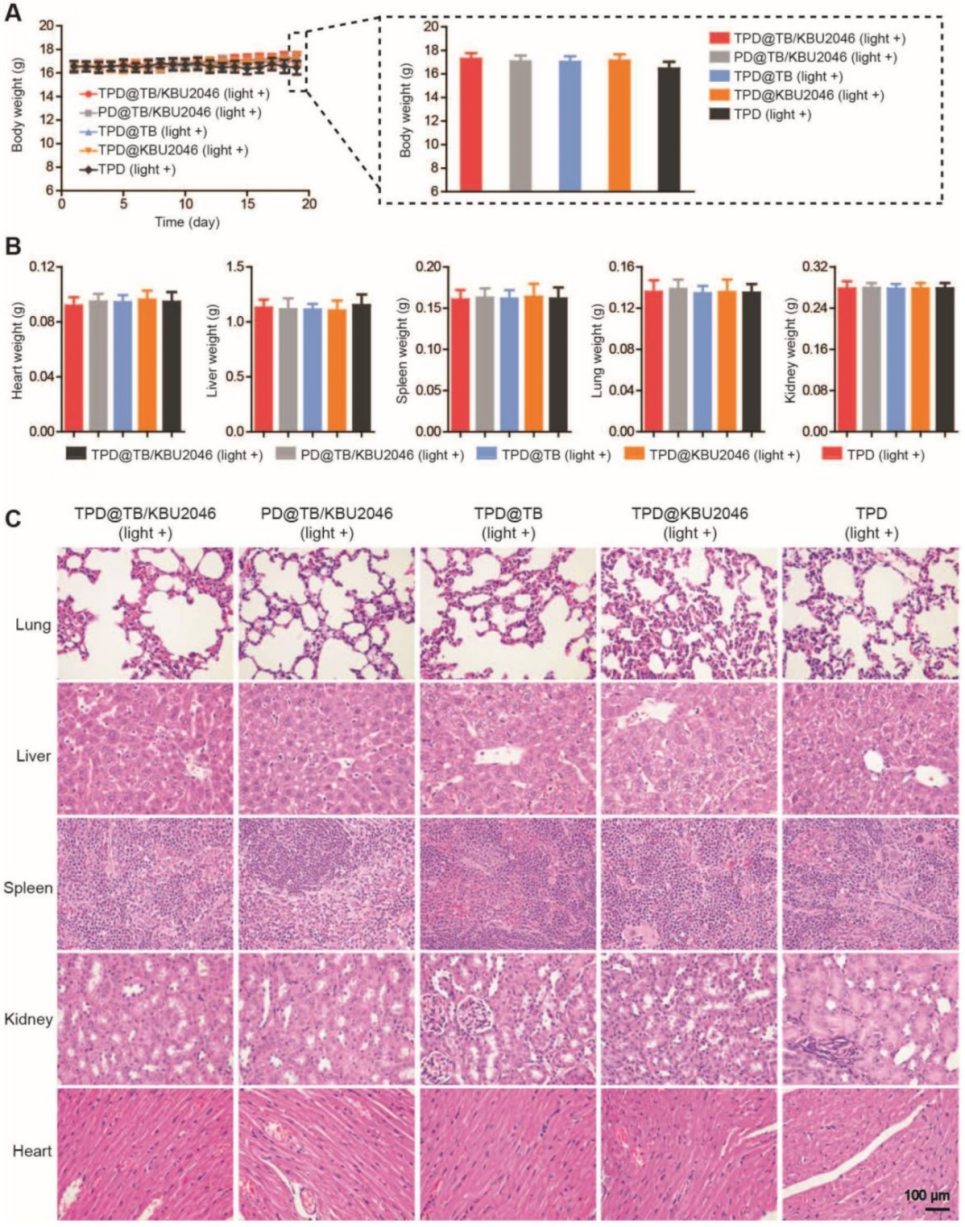
(**A**) The body weight of SKOV-3 tumor-bearing mice treated with TPD@TB/KBU2046 (light +), PD@TB/KBU2046 (light +), TPD@TB (light +), TPD@KBU2046 (light +) and TPD (light +), respectively. Irradiation intensity: 200 mW cm^-2^. Irradiation time: 20 min. Data were presented as the mean ± SD (n =5). After treated with different nanoparticles, (**B**) the weight of heart, liver, spleen, lung and kidney was measured, and (**C**) the histological changes of organs were observed by H&E staining. Data were presented as the mean ± SD (n =5).

Low toxicity or even no toxicity of biological materials is a prerequisite for the further application 57. The weight of mice did not decrease significantly, and there was no significant difference among the groups (Figure 6A). Meanwhile, the weight of the organs such as heart, liver, spleen, lung and kidney of mice in different treatment groups had no significant difference (Figure 6B). H&E staining of the organs in the different groups were also performed. As displayed in Figure 6C, there were no pathological changes in heart, liver, spleen, lung and kidney in each group after injected with different nanoparticles 58, 59. In conclusion, the above results showed that the nanoparticles had good biocompatibility in living mice and can be used for biomedical applications* in vivo*.

### Cooperation therapy between anti-growth and anti-metastasis to reduce recurrence and metastasis after surgery in orthotopic ovarian tumor model

**Figure 7:**
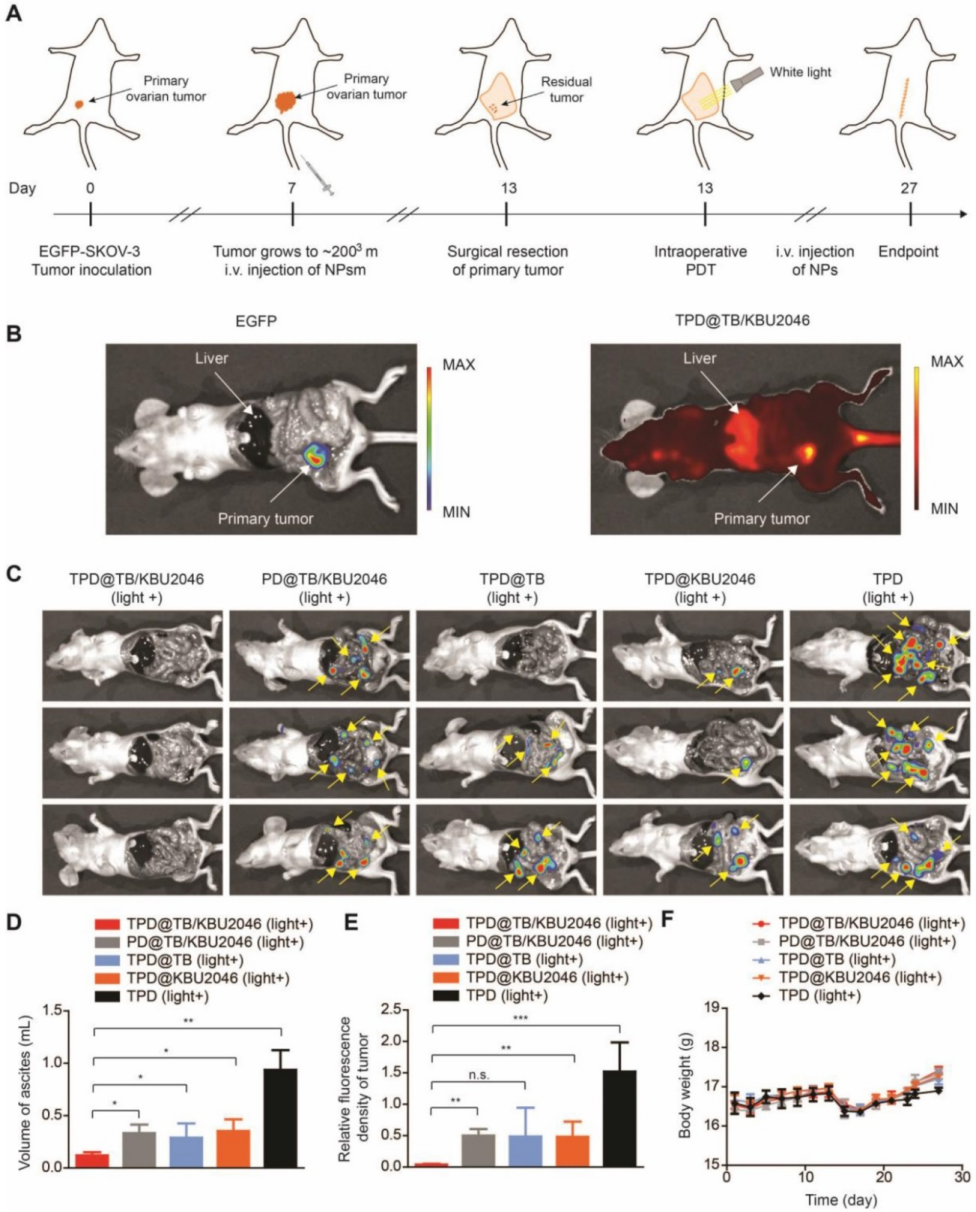
(**A**) Schematic illustrating anti-metastasis by small molecule inhibitors and anti-growth by photodynamic-AIEgens in an orthotopic ovarian cancer model. (**B**) The images of EGFP and TPD@TB/KBU2046 NPs of primary tumor *in vivo*. EGFP (E_x_=500 nm, E_m_=540 nm); TPD@TB/KBU2046 (E_x_=540 nm, E_m_=680 nm). (**C**) Images of recurrent and metastatic tumors after treatment. EGFP (E_x_=500 nm, E_m_=540 nm) (**D**) The amount of cancerous ascites after treatment. (**E**) Relative fluorescence intensity of recurrent and metastatic tumors after treatment. (**F**) Weight changes in mice during treatment. Data were presented as the mean ± SD (n =3) and analyzed by two-sided Student's t-test. n.s. not significant, * P <0.05, ** *P* <0.01 *** P <0.001.

In order to test the universality of this strategy and further improve the therapeutic effect of PDT, we used an orthotopic ovarian tumor model, combined with surgical treatment to indirectly increase the penetration depth of light, thereby improving the curative effect of PDT. Before treatment, we found that TPD@TB/KBU2046 also had a good targeting effect on orthotopic ovarian tumor (Figure 7B). 15 mice were divided into five groups: (1) TPD@TB/KBU2046; (2) PD@TB/KBU2046; (3) TPD@TB; (4) TPD@KBU2046; and (5) TPD. All the mice were treated according to the procedure shown in Figure 7A. After treatment, all mice were executed and the distribution of EGFP-SKOV-3 tumors in the abdominal cavity was detected by IVIS. Recurrence and metastasis of ovarian cancer were shown by the yellow arrow (Figure 7C). Recurrence and metastatic ovarian cancer can produce cancerous ascites, which reflect the condition of ovarian cancer to a certain extent 60. Our statistical results showed that ascites formation was almost not detected in TPD@TB/KBU2046 group (0.12 ±0.03 mL), but a large number of ascites were formed in PD@TB/KBU2046 (0.33 ±0.08 mL), TPD@TB (0.29 ±0.14 mL), TPD@KBU2046 (0.35 ±0.11 mL) and TPD groups (0.94 ± 0.19 mL), which meant that TPD@TB/KBU2046 group had the best therapeutic effect (Figure 7D). The relative fluorescence intensity of recurrent and metastatic tumors in TPD@TB/KBU2046, PD@TB/KBU2046, TPD@TB, TPD@KBU2046 and TPD groups were 0.04 ±0.01, 0.5 ± 0.11, 0.49 ± 0.46, 0.48 ± 0.24, and 1.52 ±0.46, respectively (Figure 7E). In conclusion, these results suggested that cooperative therapy between anti-metastasis by small molecule inhibitors and anti-growth by photodynamic-AIEgens could effectively inhibit the recurrence and metastasis of ovarian cancer after surgery.

## Conclusions

In conclusion, we proposed an effective self- assembled TMTP1-targeted nanoparticle entrapping AIEgen and small molecule inhibitor for image- guided PDT and anti-metastasis therapy. The targeting effect of TPD@TB/KBU2046 nanoparticles based on TMTP1 can increase the concentration of AIEgen (TB) and small molecule inhibitor (KBU2046) in the tumor site and reduce toxic effects toward normal tissues. The nanoparticles were demonstrated to exhibit superb ROS generation efficiency as well as excellent anti-metastasis capability. TPD@TB/KBU2046 can achieve cooperative treatment of anti-metastasis therapy and image-guided anti-growth for ovarian cancer both *in vitro* and *in vivo*. All in all, these findings implied that using photodynamic-AIEgens to compensate for the congenital defects of small molecule inhibitors may be a simple and efficient strategy.

## Supplementary Material

Supplementary figures and tables.
