# Supplementary material for: Cooperation therapy between anti-growth by photodynamic-AIEgens and anti-metastasis by small molecule inhibitors in ovarian cancer
Source: Theranostics. 2020 Jan 16;10(5):2385–98. doi: 10.7150/thno.41708 (PMC7019153; doi:10.7150/thno.41708)
Supplement: Supplementary file 1 — Supplementary figures and tables. [file thnov10p2385s1.pdf]

Cooperation therapy between anti-metastasis by small molecule inhibitors and anti-growth by photodynamic-AIEgens in ovarian cancer

Jun Dai [*1], Min Xu [*2], Quan Wang [2], Juliang Yang [2], Jinjin Zhang [1], Pengfei Cui [1], Wenwen Wang [1], Xiaoding Lou [✉2], Fan Xia [✉2], Shixuan Wang [✉1]

[1] Department of Obstetrics and Gynecology, Tongji Hospital, Tongji Medical College, Huazhong University of Science and Technology, Wuhan 430030, China.

[2] Engineering Research Center of Nano-Geomaterials of the Ministry of Education, Faculty of Materials Science and Chemistry, China University of Geosciences, Wuhan 430074, China.

* These authors contributed equally to this work.

✉ Corresponding author: louxiaoding@cug.edu.cn (Xiaoding Lou); xiafan@cug.edu.cn (Fan Xia); shixuanwang@tjh.tjmu.edu.cn (Shixuan Wang)

2. Supporting Figures

Figure S1

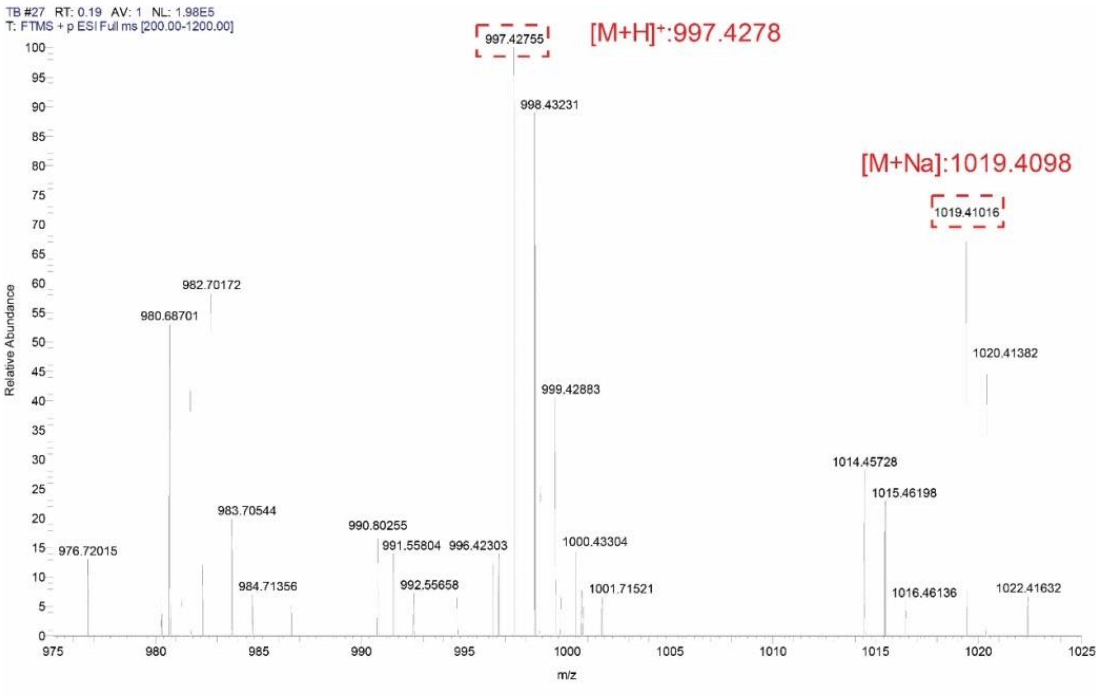

Figure S1. High resolution mass spectrum (HRMS) (MALDI-TOF) of TB, $[M+H]^+$ 997.42775 (calcd 997.4278); [M+Na] 1019.41016 (calcd 1019.4098).

Figure S2

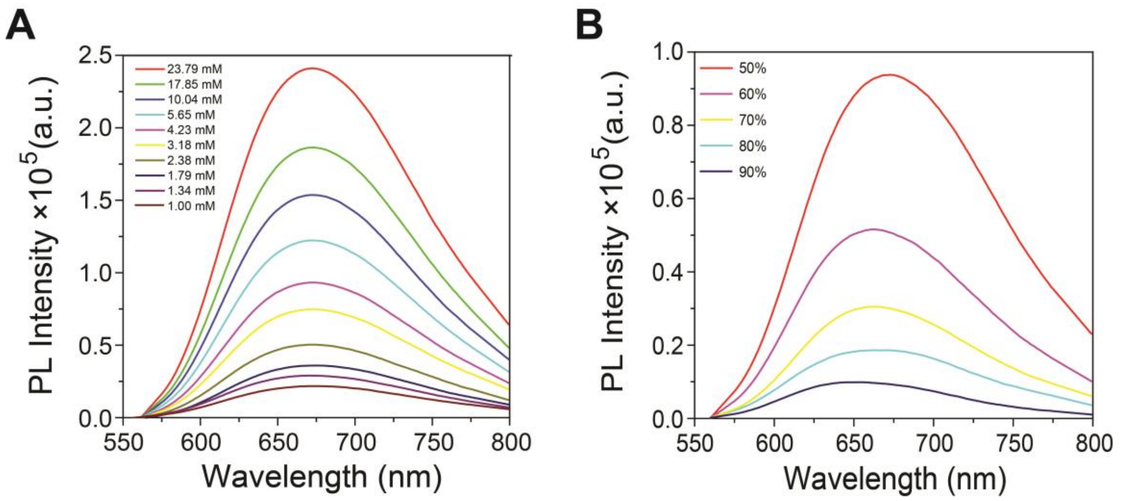

Figure S2. (A) The PL intensity of TB with different concentrations. (B) The PL intensity of TB (1 mM) in the THF/water mixtures.

Figure S3

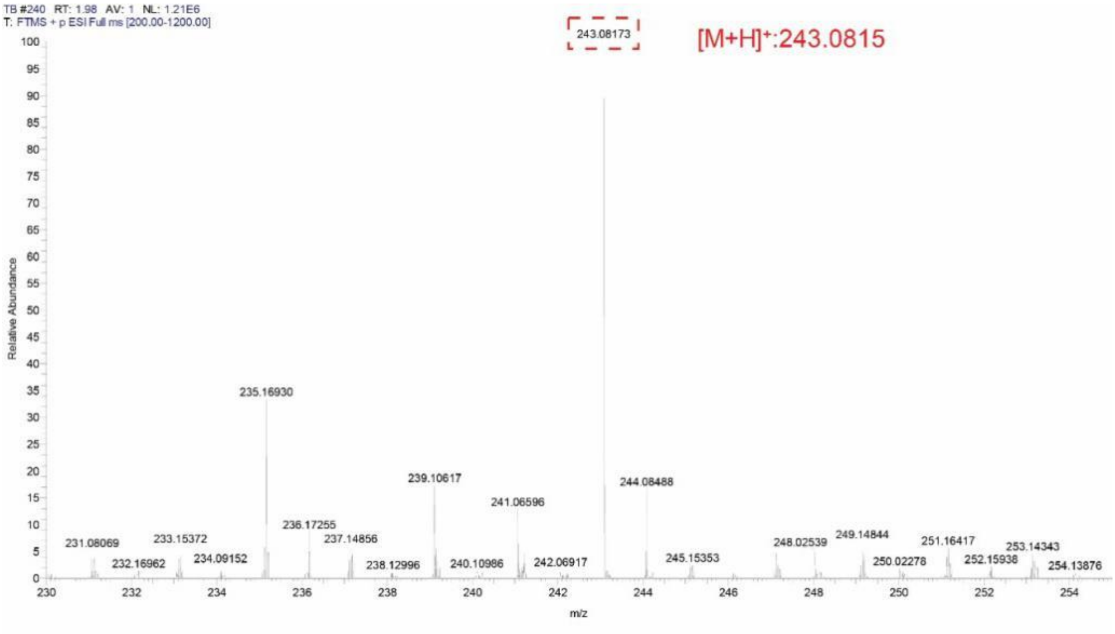

Figure S3. The HRMS (MALDI-TOF) of KBU2046, $[M+H]^+$ 243.08173 (calcd 243.0815).

Figure S4

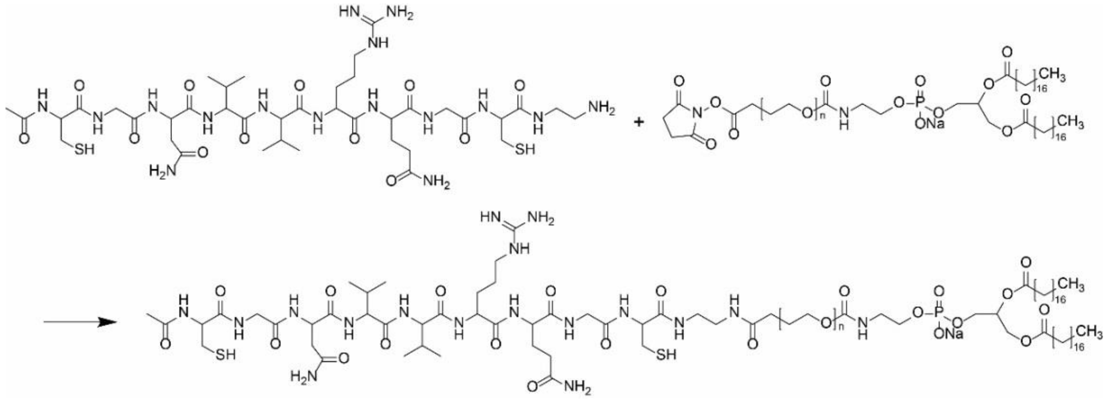

Figure S4. Synthesis of TMTP1-PEG-DSPE.

Figure S5

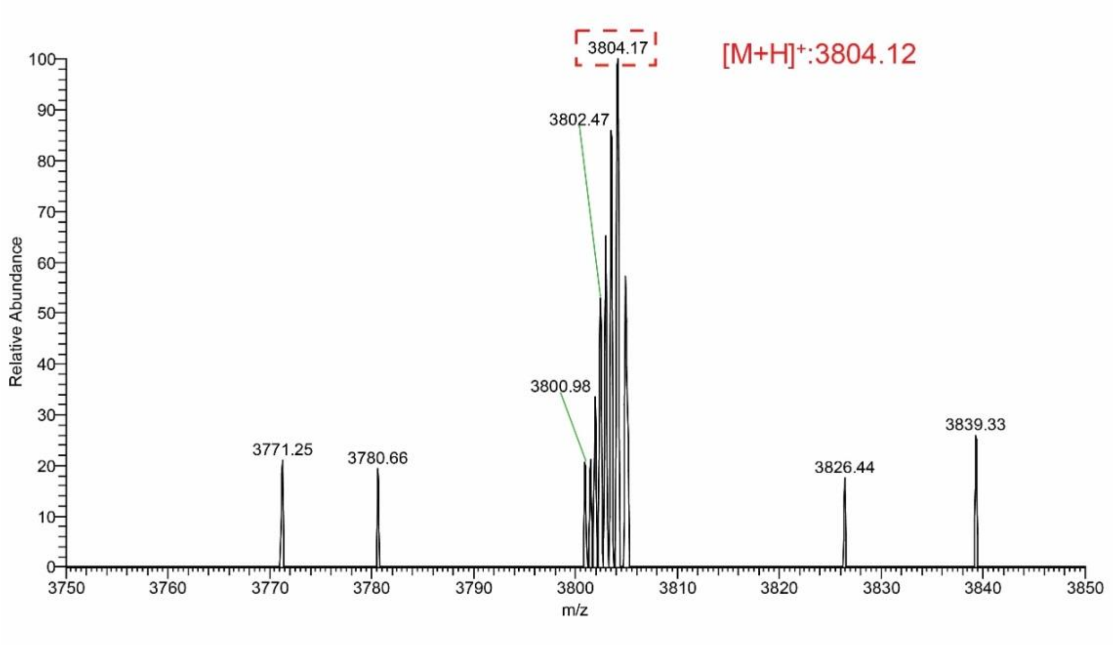

Figure S5. The HRMS (MALDI-TOF) of TMTP1-PEG-DSPE, $[M+H]^+$ 3804.17 (calcd 3804.12).

Figure S6

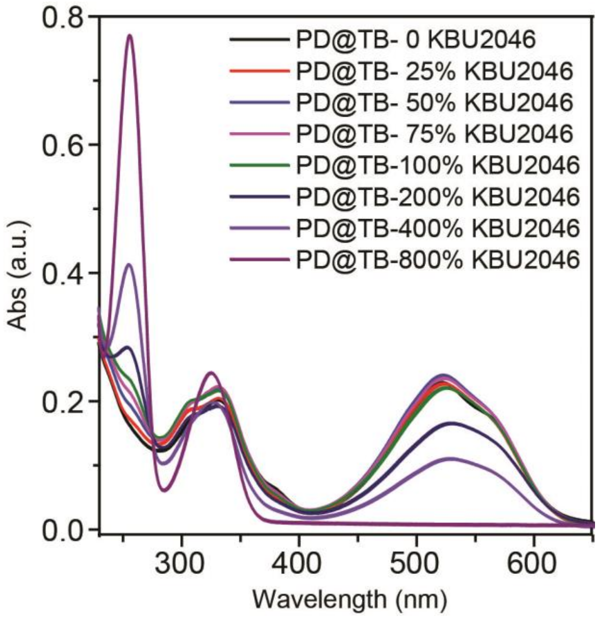

Figure S6. The drug loading capacity investigation of TPD@TB/KBU2046 nanoparticles.

Figure S7

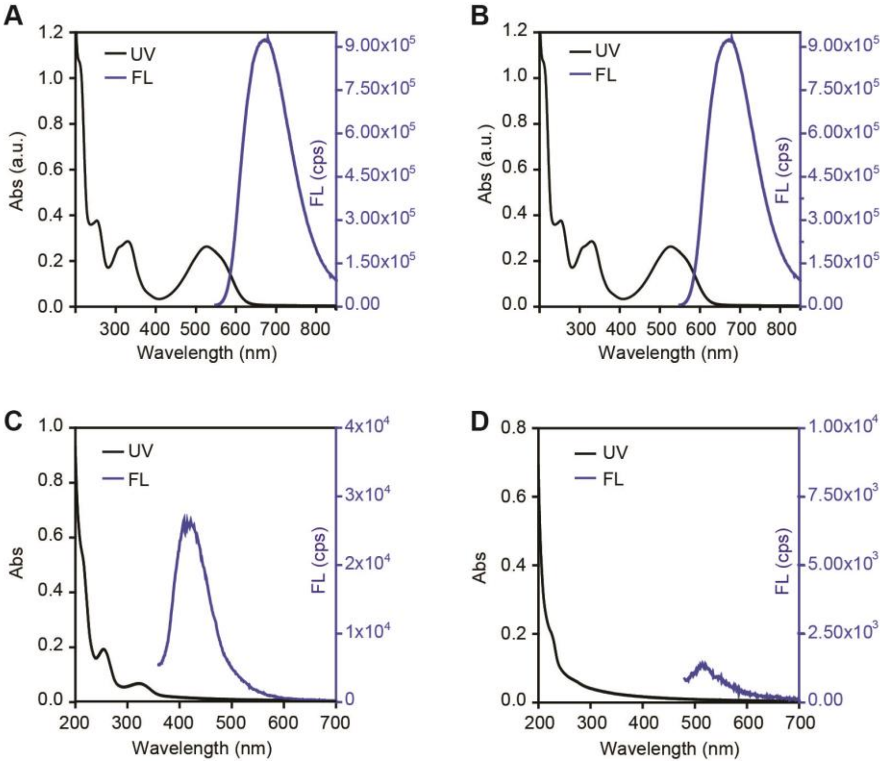

Figure S7. The UV-vis absorption and emission (E_x = 530 nm) spectrum of (A) PD@TB/KBU2046, (B) TPD@TB, (C) TPD@KBU2046 and (D) TPD nanoparticles.

Figure S8

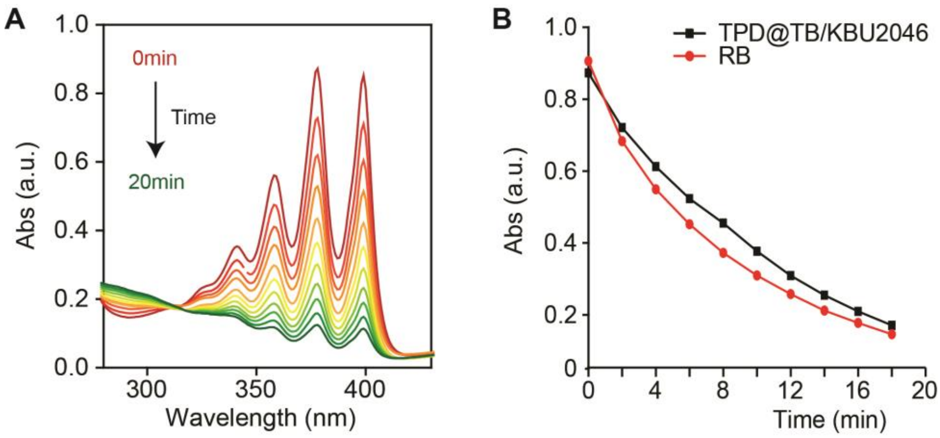

Figure S8. (A) The UV-vis absorption spectrum of the ROS indicator ABDA mixed with RB upon light irradiation (white light, 100 mW cm^{-2}). (B) The photosensitive activity of TPD@TB/KBU2046 nanoparticles and RB.
